# Recommendations for a Culturally Relevant Internet-Based Tool to Promote Physical Activity Among Overweight Young African American Women, Alabama, 2010–2011

**DOI:** 10.5888/pcd11.130169

**Published:** 2014-01-16

**Authors:** Nefertiti H. Durant, Rodney P. Joseph, Andrea Cherrington, Yendelela Cuffee, BernNadette Knight, Dwight Lewis, Jeroan J. Allison

**Affiliations:** Author Affiliations: Rodney P. Joseph, Andrea Cherrington, BernNadette Knight, Dwight Lewis, Jr, University of Alabama at Birmingham, Birmingham, Alabama; Yendelela Cuffee, New York University School of Medicine, New York, New York; Jeroan J. Allison, University of Massachusetts Medical School, Worcester, Massachusetts.

## Abstract

**Introduction:**

Innovative approaches are needed to promote physical activity among young adult overweight and obese African American women. We sought to describe key elements that African American women desire in a culturally relevant Internet-based tool to promote physical activity among overweight and obese young adult African American women.

**Methods:**

A mixed-method approach combining nominal group technique and traditional focus groups was used to elicit recommendations for the development of an Internet-based physical activity promotion tool. Participants, ages 19 to 30 years, were enrolled in a major university. Nominal group technique sessions were conducted to identify themes viewed as key features for inclusion in a culturally relevant Internet-based tool. Confirmatory focus groups were conducted to verify and elicit more in-depth information on the themes.

**Results:**

Twenty-nine women participated in nominal group (n = 13) and traditional focus group sessions (n = 16). Features that emerged to be included in a culturally relevant Internet-based physical activity promotion tool were personalized website pages, diverse body images on websites and in videos, motivational stories about physical activity and women similar to themselves in size and body shape, tips on hair care maintenance during physical activity, and online social support through social media (eg, Facebook, Twitter).

**Conclusion:**

Incorporating existing social media tools and motivational stories from young adult African American women in Internet-based tools may increase the feasibility, acceptability, and success of Internet-based physical activity programs in this high-risk, understudied population.

## Introduction

In the United States, 80% of African American (AA) women are overweight or obese, compared with 60% of white women (1). Promotion of physical activity (PA) among AA women during young adulthood when weight gain increases (2) and PA decreases (3) could be important in addressing obesity in this at-risk population.

Internet-based PA interventions have become increasingly prevalent and show promise for promoting PA. Reviews of Internet-based PA interventions report that most have been associated with positive increases in PA (4–7). However, few studies have examined the use of Internet-based approaches for the promotion of PA among AA women (7). Our review of the literature identified only 1 study evaluating the effects of an Internet-based tool among AA women. This study, conducted by Pekmezi et al (8), evaluated the feasibility of an Internet-based PA intervention among a subset of AA women enrolled in a larger randomized controlled trial (9). Results showed a significant increase in PA and favorable outcomes for the acceptability and feasibility of the Internet-based approach (8). However, the program was designed for middle-aged to older women and was not culturally relevant.

Research suggests barriers to PA among middle-aged and older AA women include lack of social support, lack of time, hair care concerns, and body image concerns (10–12). However, barriers in young adult AA women may differ from older women. Few studies have conducted a focused examination of barriers to PA among young adult AA women or developed PA programs targeting young adult AA women. The lack of PA programs targeting this high-risk population represents a missed opportunity.

We sought to address 2 important gaps in the literature: 1) identification of barriers to PA in overweight and obese young adult AA women and 2) identification of features to include in a culturally appropriate Internet-based PA intervention to promote PA in overweight and obese young adult AA women.

## Methods

The primary objective of this study was to conduct a mixed-method qualitative assessment by using nominal group technique (NGT) (phase 1) and traditional focus group technique (phase 2) to determine what features should be included in an Internet-based tool to promote PA in overweight and obese young adult AA women. This study was conducted at the University of Alabama at Birmingham (UAB). Data were collected during 2010 and 2011. All study procedures were approved by the university’s institutional review board.

### Study participants

Overweight and obese AA women were recruited via fliers and face-to-face contact. Eligibility criteria were 1) self-identified as AA, 2) aged 19 to 30 years; 3) enrolled in the university; and 4) body mass index of 25 kg/m^2^ or higher. University staff enrolled in classes were eligible to participate. Participants provided informed consent and received $20 for participation. Participants in phases 1 and 2 completed demographic questionnaires. Height (in meters) and weight (in kilograms) were measured by trained study staff and used to calculate body mass index.

### Phase 1

The aim of phase 1 was to identify website features that young overweight and obese AA women desire in a culturally relevant PA promotion website.

We conducted 2 NGT sessions led by 2 female AA facilitators. Sessions were audio recorded and transcribed verbatim. NGT facilitates balanced participation among participants (13) and permits qualitative data to be transformed into a structured rank list (14). This method has been previously used in studies to generate feedback to develop health promotion programs targeted to AAs (15,16).

In the NGT session, participants (n = 13) logged on and reviewed the PA promotion sections of a commercial website, www.sparkpeople.com. This website was chosen because of its popularity among lay individuals. Sample features on the website include exercise demonstration videos, motivational tips, and exercise tracking. After viewing designated sections of the website, participants were asked, “What features of the website are most important to include in a physical activity promotion website for overweight and obese African American women?” Participants were then instructed to share responses in a round-robin fashion with the group. Responses were displayed on a board for all participants to view. Participants were allowed to ask for and provide clarification of responses. Last, participants were instructed to identify and rank their top 3 responses (3 = most important and 1 = least important).

Two investigators independently examined the responses ranked 3, 2, and 1 from each participant. Responses with similar themes were grouped based on expert team consensus. Top ranked themes were used in focus group guide development for phase 2.

### Phase 2

The aim of phase 2 was to confirm and verify themes that emerged in the NGT sessions and to gather additional information that may have been missed during the NGT process. A secondary aim of this phase was to query participants about barriers to PA.

Two focus groups were moderated by 2 trained, AA female research assistants (1 moderator and 1 note taker). Focus groups were audio recorded and transcribed verbatim. Participants were queried about barriers to PA and about what features they would recommend for inclusion in a culturally relevant Internet-based PA promotion tool. The moderator used a focus group guide to lead the discussion. The guide included an overview of the study and questions to guide the discussion. Transcripts were independently reviewed for major themes by 3 PhD-level evaluators. Evaluators met to discuss findings and finalize recommendations on major/repetitive themes that emerged from the data.

## Results

Twenty-nine women participated in NGT sessions (n = 13) and traditional focus groups (n = 16) (Table 1). On average, participants had a body mass index of approximately 30 kg/m^2^ and most were not married. There were no significant differences in demographic characteristics between the women who participated in phase 1 and phase 2. Four women participated in both the NGT and focus group sessions. This process, known “member checking” (17), was used to verify and confirm responses obtained from the phase 1 NGT sessions.

**Table 1:** Characteristics of Participants in Assessments of an Internet-Based Tool to Promote Physical Activity, Alabama, 2010–2011^a^

Characteristic	Overall Sample	Phase 1: Nominal Group Technique Session, n = 13	Phase 2: Focus Group Sessions, n = 16
Age, y, mean (SD)	23.9 (2.6)	24.5 (3.1)	23.9 (1.9)
Body mass index, kg/m^2^, mean (SD)	31.1 (5.5)	30.8 (6.2)	30.7 (5.1)
**Race/ethnicity**			
Non-Hispanic black	28	13	15
No response	1	0	1
**Marital status**			
Single	25	10	15
Married	3	2	1
Marriage-like relationship	1	1	0
**Highest degree obtained**			
High school diploma or GED	12	4	8
Associate	1	0	1
Bachelor’s or higher	16	9	7
**Highest degree obtained by parents**			
Less than high school diploma	2	1	1
High school diploma or GED	8	3	5
Associate	2	1	1
Bachelor’s or higher	16	8	8
No response	1	0	1
**Annual household income, $**			
≤10,000	12	2	10
10,001–20,000	4	3	1
20,001–30,000	3	1	2
30,001–40,000	4	4	0
>40,000	5	2	3
Don’t know	1	1	0

Abbreviation: SD, standard deviation; GED, General Educational Development certificate.

a Data presented are number unless otherwise indicated.

### Phase 1 outcomes

Exercise demonstration videos, followed by motivational tools or inspirational stories about maintaining PA, received the highest rankings (Figure). The overall rank score and the summed votes for each feature resulted in identical outcomes for the order of inclusion of features in the tool.

**Figure:**
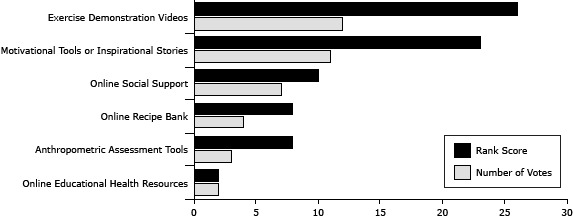
Scores for features of a website that are most important to include in a physical activity promotion website for overweight and obese African American women, Alabama, 2010–2011. Each participant ranked features from 1 to 3 (3 = most important and 1 = least important). Participants could use each rank only once. Rank score represents the sum of all ranks for each feature. **Table:** 

Feature	Rank Score	Number of Votes
Exercise demonstration videos	26	12
Motivational tools or inspirational stories	23	11
Online social support	10	7
Online recipe bank	8	4
Anthropometric assessment tools	8	3
Online educational health resources	2	2

### Phase 2 outcomes

Table 2 illustrates barriers to PA identified by our sample of young adult AA female college students. Barriers to PA included lack of time, social stigma or insecurity, hair care maintenance, and economic or environmental concerns.

**Table 2:** Barriers to Physical Activity Identified in Focus Groups Discussing Recommendations for a Culturally Relevant Physical Activity Promotion Website for African American Women, Alabama, 2010–2011

Barrier	Example Quote
Hair concerns	• I’m trying to work out now, and I’m finding that I can’t keep a hairstyle. (Focus group 2, participant 6) • Girl, I just got my hair done, and I’m not going to sweat it out because I’m going out this weekend. (Focus group 1, participant 2)
Social stigma or insecurity	• I don’t like to go the gym because I feel so out of place. . . . I look at the other girls like, “Why are you here? . . . How small do you want to be?” (Focus group 1, participant 4) • It’s about being shameful about not knowing how to work the equipment. (Focus group 1, participant 3)
Lack of social support	• I’m much more likely to do something if I have somebody that I can talk to or work [out] with. (Focus group 2, participant 1) • Not having a buddy! [when asked to describe barriers to physical activity] (Focus group 2, participant 1)
Lack of time	• You don’t want to go [to the gym] because, “Oh, I have to study for that test.” (Focus group 2, participant 9) • We’re all students so schoolwork in itself; plus, some are employed; plus, life itself is demanding . . . so basically schedule in itself. (Focus group 1, participant 1) • Lack of time. [when asked to describe barriers to physical activity] (Focus group 2, participant 4)
Lack of knowledge	• Me personally, it’s not knowing how to work the machinery. (Focus group 1, participant 3) • As for me [in references to barriers to physical activity], it would just be [knowing] different types of exercises . . . maybe like modified, something that is not too strenuous starting out. (Focus group 2, participant 2)
Lack of motivation	• I have problems keeping myself going to the gym. (Focus group 1, participant 6) • Lack of motivation. [when asked to describe barriers to physical activity] (Focus group 2, participant 3) • At the end of the day, work out is that last thing you want to do. (Focus group 2, participant 4)
Economic or environmental constraints	• I would like to do it [exercise] at night or after work . . . [but] I live in Southside and we can’t go running there at night [because of safety concerns]. (Focus group 1, participant 5) • You can’t afford to get a personal trainer. (Focus group 1, participant 3)

Table 3 presents the major focus group themes, sample quotes from participants and potential Internet-based features to include in an Internet-based PA intervention. NGT themes confirmed in phase 2 focus groups included exercise demonstration videos, motivational stories, and online social support. New themes that emerged included personalization of website pages, inclusion of diverse body images on websites and in videos, and provision of tips on hair care maintenance of natural and relaxed hair during PA.

**Table 3:** Qualitative Themes and Recommendations Identified in Focus Groups Discussing a Culturally Relevant Physical Activity Promotion Website for African American Women, Alabama, 2010–2011

Theme	Sample Quote	Internet-Based Application
Personalization	I think kind of along the lines of the Facebook idea. Just give the people some kind of blog or some kind of newsfeed. . . . Maybe not [just] someone who *looks like you* on the site, but maybe just a picture *of you*. That way, maybe you would have a photo album saying, “Look at my success.” You could put up what you feel like has helped you along the way — an area of inspiration. (Focus group 1, participant 6)	Provide personalized home page with the ability to upload photographs, upload videos, and the ability to post and receive messages

Inclusion of diverse body images	I would like someone with a little tire [*motions toward her waistline*], some thighs [*points to her thighs*]. Just someone I could look at and say, “Maybe there *is* a little support system for me if they look like me.” [Then] I [would] feel automatically drawn to it. A little bit more of my guard is down. I would feel like I could actually identify with the people who are on there. (Focus group 1, participant 5)	Provide diversity of body types in images displayed on the website
	• [You] need to see *real* people that [are] about your size and think, “If she can do it, I can do it!” (Focus group 2, participant 1) • [Include] Pictures of someone who looks like me. [*Everyone laughs and agrees.*] I don’t want somebody *extra* skinny trying to teach me to do something that I can’t get my body to do what they’re doing. I know a lot of times you see [on] TV or a video [and] they’ve got the lady and she’s got her little sports bra and little shorts. I mean, I can’t wear that to the gym! I want somebody that’s got a T-shirt and sweat pants on like I’m going to wear! That’s going to make me feel more comfortable and more motivated to exercise. (Focus group 2, participant 10)	Incorporate instructors with different body types and comfortable workout clothing into exercise demonstration videos

Online social support	I think my biggest problem is timing my workouts with my friends. It’s not that they don’t want to go to gym. Maybe [the site] could have blocked categories like, Could you go from 9:00 to 12:00? (Focus group 1, participant 3)	• Online exercise scheduling • Blogs: facilitate discussion of successes and struggles with physical activity
	Me personally, I don’t think I can get a group — especially black girls — together right now to go work out today because most of my friends are like, “*Girl, I just got my hair done, and I’m not going to sweat it out because I’m going out this weekend*.” There are so many different excuses. If I found a group of people online who are willing to meet me every Wednesday at the same place to work out and share the same problems (such as trying to lose weight) that may be beneficial as a kind of support. (Focus group 1, participant 2)	Message boards: facilitate discussion of physical activity goals, opportunities outside of school and work, hair care tips for hair maintenance during physical activity

Motivation	I think that having a strong motivational section is very important. Motivation could be anything — photo albums with not just before-and-after pictures but also the “in-between pictures.” I want to see the process. I think that would keep folks motivated. (Focus group 1, participant 6)	Videos of stories of “real” women sharing their physical activity journeys
	Having target goals like “where you are today” or “this is your target goal for today.” Or if you don’t log in for a couple of days, something like, “Well, since you missed your target day, this is your target *now*.” (Focus group 1, participant 3)	Goal setting feature

Physical activity: desire for exercise instruction	Me personally, I used to not know how to work the equipment. So, I would just stick with what I know how to do and that may not work for the part of my body where I need to get rid of some things. [*Everyone laughs and agrees.*] I guess it goes back to insecurity. It’s not worrying about the other people around me; it’s being shameful about not knowing how to work the machinery. And then you can’t afford to get a personal trainer. So, if you could have guidance or personal training or something like that. (Focus group 1, participant 3)	Online personal training
	You could have a section where they show you how to do stuff at home. Something where you don’t *have* to go to the gym. Squats or *anything* you can do at home. [You could give examples of] exercises you could do at home. Say it’s a rainy day; stretches and things that would still work you out a bit. (Focus group 1, participant 4)	• Exercise demonstration videos • Exercise tips for outside of gym (eg, home)

Physical activity: desire for tracking	• A log that — well, I guess like an exercise routine. You could write down how much you need to do or what areas to focus on for a particular day in time. (Focus group 2, participant 2) • Maybe a [body mass index] calculator. (Focus group 2, participant 3) • Something to record your progress. Like if you’re [aiming] for a particular goal. (Focus group 2, participant 4)	Exercise tracking

Nutrition	I would say a food diary. Sometimes you don’t realize how much you eat until you write it down. (Focus group 2, participant 1)	Diet tracking
	Another good thing, like what [another participant] said — I went to Burger King yesterday and (they said) that you could get a Whopper Jr., but to eliminate 20% of the calories and I think 40% of the fat, or something like that, just don’t put the mayo on it. Just give them tips like that. Like, “Just take this off your sandwich and take that off your sandwich and it can save you so many calories.” (Focus group 2, participant 5)	Fast food alternatives
	• Also, maybe things that you already have in your refrigerator that you can cook differently. Maybe instead of frying it, you could grill it or bake it. (Focus group 2, participant 9) • Food options. Things that you can cook or a way to prepare your foods so that they wouldn’t be so bad. . . . [Provide examples of] quick meals that can be healthy. (Focus group 2, participant 5)	Healthy recipes with a focus on quick preparation

Health and beauty applications	This is kind of far-fetched, but what about hair tips? I’m trying to work out now, and I’m finding that I can’t keep a hairstyle. So maybe some hair care or hair tips. (Focus group 2, participant 6)	Hair care maintenance application

Regarding the desire for personal profiles, participants requested the ability to upload personal information such as pictures, hobbies, and quotes. One participant stated, “It would be helpful to see, maybe not [just] someone who *looks like you* on the site, but maybe just a picture *of you*” (Focus group 1, participant 6). They also requested the ability to send and receive messages from friends via a website. Participants also wanted to upload and share videos about their experiences with PA as well as share videos, blogs, and message posts about school, work, and family.

Women emphasized the need for the inclusion of diverse body types on websites, requesting pictures of “real women” similar to themselves in size and body shape. One participant said, “I would like someone with a little tire [*motions toward her waistline*], some thighs [*points to her thighs*]. . . . [Then] I [would] feel automatically drawn to it” (Focus group 1, participant 5). Another participant echoed,

“I don’t want somebody *extra* skinny trying to teach me to do something that I can’t get my body to do what they’re doing. I know a lot of times you see [on] TV or a video [and] they’ve got the lady and she’s got her little sports bra and little shorts. I mean, I can’t wear that to the gym! I want somebody that’s got a T-shirt and sweat pants on like I’m going to wear! That’s going to make me feel more comfortable and more motivated to exercise” (Focus group 2, participant 10).

Participants desired the inclusion of motivational stories in the form of narratives depicted in pictures and videos. They highlighted the importance of portraying “real women” relaying stories of triumphs and struggles with PA. One said, “Motivation could be anything — photo albums with not just before-and-after pictures but also the ‘in-between pictures.’ . . . I think that would keep folks motivated” (Focus group 1, participant 6).

Participants consistently endorsed the incorporation of social media sites into PA promotion programs for young adult AA women. They suggested pairing an Internet-based program with existing social networking sites (eg, Facebook, Twitter, Instagram, Tumblr) to facilitate the creation of new networks among other AA women interested in PA. They also suggested including blogs and message boards to facilitate discussions of topics regarding body shape, and how to dress for your current size emerged as a major theme.

Another theme that emerged was a request for an online forum for hair care maintenance tips for natural and relaxed hair. Hair care maintenance emerged during the discussion of barriers to PA and continued during the discussion of potential features for inclusion in a website. Participants suggested online forums via message boards, blogs, Facebook, and links to magazines with tips for hair care for AA women maintaining an active lifestyle.

## Discussion

This study used a mixed-method approach combining NGT with focus groups to elicit recommendations from young overweight and obese AA women regarding features to include in a culturally relevant Internet-based PA promotion tool. Themes that emerged in both NGT and focus groups included exercise demonstration videos, motivational stories, and online social support. In confirmatory focus groups, participants requested inclusion of motivational stories featuring “real women” similar to themselves and online social support. New themes emerging from focus group sessions included personalization of website pages, inclusion of diverse body images on websites, provision of tips for AA hair care maintenance during PA, and incorporation of social media into culturally relevant programs as vehicles to create new social networks for AA women pursuing active lifestyles.

Lack of social support has been well documented as barrier to PA in older AA women (11). Older AA women who successfully maintained PA identified social support as key to their success (18). However, to our knowledge, this is the first study documenting the role of lack of social support for PA among young adult AA women. Further, to our knowledge, this is the first study to highlight the potential role of social media to promote social support of PA in this population. Our study also highlights the desire among young adults to form new social networks as a source of social support for PA.

Young adults aged 18 to 29, including AAs, use social media at high rates to create and maintain social networks (19). Data from the Pew organization indicate that Twitter, Facebook, Tumblr, and Instagram are used more frequently by adults aged 18 to 29 years than any other age group (19). Furthermore, Twitter and Instagram are used more frequently by AAs than whites, and Facebook and Instagram are used more frequently by women than men (19). Thus, using social media in Internet-based PA efforts may be beneficial to promote PA among young AA women, given that this appears to already be incorporated into the culture of young adult AA women.

Participants also requested the incorporation of personal profile pages with features similar to existing social media sites such as Facebook and MySpace. In a study conducted by Napolitano et al (20), Facebook was used in combination with text messaging to deliver a weight-loss intervention over 8 weeks to college students. At 8 weeks, participants receiving the Facebook intervention plus text messaging had significantly greater weight loss compared with those receiving Facebook only and the waiting list control (both *P* values < .05). Additional studies are needed to further explore the role of social media and online social networking in the promotion of PA among overweight and obese young adult AA women.

Narrative storytelling is an application that may combine aspects of personalization and online social support into a culturally relevant tool that can be disseminated via the Internet. Storytelling elicits, captures, and packages powerful behavior-changing stories in participants’ own voices. The process is based on a solid conceptual framework (21) and has the power to directly and effectively confront the participant’s perceptions and cultural experiences. Narrative storytelling has a long tradition in maintenance of health and wellness globally. It has improved hypertension control among low-income AAs receiving care in an inner-city hospital in Alabama (22). Future research should explore the role of storytelling in the promotion of PA among young adult AA women through Internet-based technology.

The importance of including diverse body types on websites and in videos also arose as a major theme in this study. Most research thus far on AA women and body image references AA women’s preference or tolerance for larger body types (23,24). Among our participants the discussion of body image did not focus on endorsing a larger body type. Rather, the discussion focused on the importance of including a variety of body types in PA websites and videos to provide encouragement to overweight and obese women that an active lifestyle is attainable. To our knowledge this is the first study to document young AA women’s preferences for inclusion of diversity of body types in PA promotion sites and videos.

Having features on hair care maintenance for relaxed and natural hair was also suggested. Hair care has been consistently cited as a barrier to PA among AA women (11). However, we were unable to identify a PA promotion program that addressed this issue. Further research is needed to determine what Internet platforms are best to deliver these applications.

Study limitations include enrolling a small sample of women from a single university. Results may not be generalizable to young adult AA women not enrolled in a university or women enrolled in other universities (eg, 2-year schools, historically black colleges). We also acknowledge that conducting member checking may have introduced some bias into the focus group results given that the participants who contributed to both NGT and focus groups were not queried separately from those who participated in focus groups only.

Despite these limitations, this study provides an important initial step in understanding what features are important to incorporate in a culturally relevant Internet-based PA promotion tool targeted to young adult overweight and obese AA women. We do acknowledge that not all of the features endorsed by the young women are specific to culture. For example, the request to include online social support is not culture-specific. However, incorporating current and future social media into culturally relevant Internet-based interventions represents an opportunity to leverage technology to encourage PA change in this high-risk, underserved, understudied population.

This study contributes to the field in multiple ways. First, few Internet-based studies have conducted formative assessments before development (25). Most such studies use a “top-down approach,” drawing on experts and prior literature to inform website development (25–27). Programs that do not include participant input may fail to incorporate the unique social and cultural contexts of the target population, which may in turn impair the participants’ willingness and ability to transform their behavior. The use of qualitative assessments in young adult AA women may enhance the receptivity, acceptance, and salience of PA interventions by using information gathered directly from the participants on how to address barriers to PA that have been uniquely identified in this population (12,23,24,28,29). This approach also may provide information necessary to develop programs that are developmentally appropriate. Future studies are needed to develop and assess culturally relevant Internet-based interventions for PA promotion among overweight and obese AA women that leverage state-of-the-art technologies including social media to disseminate dynamic interventions that are reproducible and sustainable.
